# Cryptic clues from an acute abdomen: a case report of peritonitis turning into disseminated cryptococcosis

**DOI:** 10.1186/s12879-026-13871-w

**Published:** 2026-07-16

**Authors:** Archith Boloor, Swarnava Porey, M. V. Ashwini, Vivek K. Koushik

**Affiliations:** 1 Department of Medicine, Kasturba Medical College Mangalore, Manipal Academy of Higher Education, Manipal, India; 2 Department of Medicine, Kasturba Medical College Mangalore, Manipal Academy of Higher Education, Manipal, India; 3 Department of Cardiology, Kasturba Medical College, Manipal Academy of Higher Education, Manipal, India; 4 Department of Nephrology, Prashanth Hospitals, Kolathur, Chennai, Tamil Nadu 600110 India

**Keywords:** Cryptococcosis, *Cryptococcus neoformans*, HIV, Peritonitis, Acute abdomen, Disseminated fungal infection, HIV and co-infections

## Abstract

**Background:**

Cryptococcosis is a life-threatening opportunistic fungal infection that primarily affects immunocompromised individuals, most commonly presenting as meningitis. Peritoneal involvement is exceedingly rare and can closely mimic common acute surgical conditions, often resulting in delayed diagnosis and treatment. This case describes an unusual presentation of disseminated cryptococcosis as acute peritonitis with subsequent progression to cryptococcal meningitis; underscoring the diagnostic challenges posed by atypical intra-abdominal infection.

**Case presentation:**

A previously healthy female homemaker in her late 20s presented with a 20-day history of intermittent low-grade fever, followed by 7 days of right lower abdominal pain and multiple episodes of non-projectile vomiting. Clinical examination revealed right iliac fossa tenderness with guarding and rebound tenderness, suggestive of acute appendicitis with perforation. Her pre-operative laboratory work-up revealed HIV positivity and emergency laparotomy with open appendicectomy and drainage of pelvic abscess was performed. Histopathological examination demonstrated granulomatous inflammation with encapsulated yeast forms consistent with *Cryptococcus neoformans*, later confirmed by special fungal stains. On postoperative day 3, the patient developed headache and neck stiffness. Subsequent evaluation identified disseminated cryptococcosis with cryptococcal meningitis, mesenteric lymphadenitis, and urinary tract involvement, alongside newly diagnosed advanced HIV infection with CD4 cell count of 98 cells/mm³. The patient received amphotericin B-based induction antifungal therapy followed by prolonged fluconazole consolidation and maintenance therapy. Antiretroviral treatment was initiated in a deferred manner after stabilisation to avoid IRIS related complications. Clinical improvement was observed with resolution of symptoms and significant immunological recovery during follow-up with no residual complications.

**Conclusions:**

This case illustrates an exceptionally rare presentation of disseminated cryptococcosis manifesting initially as acute peritonitis and later progressing to central nervous system involvement. To our knowledge, this is the first reported case where peritonitis precedes systemic involvement and meningitis in cryptococcosis clinically in HIV positive patients. It emphasizes the importance of maintaining a high index of clinical suspicion for opportunistic fungal infections in atypical or non-resolving intra-abdominal infections, particularly in patients with undiagnosed immunosuppression. Early histopathological recognition, timely antifungal therapy, and coordinated management of underlying HIV infection are critical for improving outcomes.

**Supplementary Information:**

The online version contains supplementary material available at https://doi.org/10.1186/s12879-026-13871-w.

## Introduction

Cryptococcosis is an invasive fungal infection, that predominantly affects immunocompromised individuals, especially in advanced HIV infection with CD4 count less than 100cells/microL. Despite widespread antiretroviral therapy, cryptococcal meningitis remains a major global health problem and a leading cause of AIDS-related mortality worldwide [1]. In contrast, peritoneal involvement is an exceptionally rare manifestation in HIV-positive patients and is most commonly reported in those with cirrhosis or on peritoneal dialysis [2]. Cryptococcal peritonitis often closely mimics spontaneous bacterial peritonitis with nonspecific symptoms, and initially negative ascitic fluid studies; leading to delayed diagnosis [3]. 

## Case report

A female homemaker in her late 20s, presented with right lower abdominal pain for 7 days and multiple episodes of non-projectile, non-bilious vomiting one day prior to admission. She had a 20-day history of low-grade intermittent fever. The abdominal pain was sharp, aching, localized to the right lower quadrant, without radiation or diurnal variation and worsened with ambulation. She reported associated anorexia and sleep disturbance. There were no urinary, gastrointestinal, respiratory complaints or history of weight loss. Her menstrual history was also regular, and her family history was unremarkable. She had no comorbidities and denied any habits. On physical examination, she was found to have rebound tenderness in the right iliac fossa with guarding. The prolonged episode of fever preceding abdominal symptoms prompted consideration of a broad differential diagnosis, including systemic, gynaecological, and urinary causes, which in reproductive-age women can often mimic an acute surgical abdomen.

Normal serum β-hCG levels ruled out any ectopic pregnancies, but her pre-operative serology revealed HIV positivity. Since she was hailing from a region endemic to Leptospirosis, acute febrile illness profile was done to rule out any underlying infections. The absence of raised intracranial pressure signs, headache, focal neurological deficits and a preserved Glasgow-Coma-Scale score made primary meningeal infection unlikely. In the absence of genitourinary symptoms, appendicitis, with possible perforation was considered as the leading differential diagnosis. Gastroenteritis was deemed less likely due to the absence of diarrhoea and the localisation of pain to the right lower quadrant.

These findings were corroborated by ultrasonography abdomen, which revealed free fluid in the pelvis, and probe tenderness in the right iliac fossa. In view of suspected intra-abdominal sepsis, the patient underwent emergency laparotomy with open appendicectomy and drainage of a pelvic abscess.

During surgery, the pus was collected and sent for culture and sensitivity; omental biopsy and appendix specimen was sent for Histopathological examination. Histopathology revealed transmural granulomatous inflammation with fungal spores (Supplementary Figure-A). For confirmation, special stains Grocott methenamine silver (GMS) and Periodic Acid-Schiff (PAS) were positive showing oval/round yeast cells with polysaccharide capsule with a prominent unstained pericellular halo corresponding to the polysaccharide capsule suggestive of cryptococcal appendicitis (Supplementary Figure-B, C). Diagnosis of Histoplasmosis was excluded as mucicarmine stain was negative. Thus, microbiological and histopathological examination demonstrated the presence of cryptococcal spores, confirming a diagnosis of cryptococcal appendicitis.

Urine routine showed 2 to 3 pus cells and yeast like budding cells. PAS stain done in urine sample was also positive indicating cryptococcuria (Supplementary Figure-D).

On post-operative day 3, the patient developed headache. This coincided with our next goal to evaluate for and limit any systemic infection following the histopathological results. Examination revealed neck stiffness without focal neurological deficits, and fundus examination was normal. CT brain was unremarkable. Lumbar Puncture and Cerebrospinal Spinal Fluid (CSF) analysis revealed a total cell count of 50, all lymphocytes. CSF-CBNAAT (Cartridge-Based Nucleic Acid Amplification Test) was negative for Tuberculosis. However, India Ink staining was positive and *Cryptococcus neoformans* was grown after 48 h of incubation, confirming the diagnosis of cryptococcal meningitis. CD4 count was 98 cells/mm³. MRI Brain with contrast showed cryptococcomas. CECT abdomen done in view of persistent drain output on post operative day 9 showed multiple enlarged mesenteric lymph nodes but showed no renal involvement. CECT Lung came back normal with no signs of disease or mediastinal lymph node involvement. Subsequently, with the presence of cryptococcuria, disseminated cryptococcal infection was confirmed.

### Treatment and outcome

Patient was started on induction therapy of intravenous amphotericin B deoxycholate 50 mg (1 mg/kg) daily for 14 days, along with mannitol and dexamethasone. Cotrimoxazole double strength tablet once daily was started for Pneumocystis pneumonia prophylaxis. Due to unavailability of flucytosine, amphotericin B deoxycholate was followed by high-dose oral fluconazole 1200 mg daily for 14 days.

Repeat Lumbar Puncture done after 14 days of amphotericin B and fluconazole treatment showed persistent India Ink positivity, but the patient showed progressive clinical improvement. Following up, fluconazole 800 mg over 2 doses daily was continued for 8 weeks.

The patient was put on fluconazole continuation therapy 200 mg daily for 12 months and then finally stopped. Anti retroviral therapy (Tenofovir disoproxilfumararte 300 mg-Lamivudine 300 mg-Dolutegravir 50 mg regimen) was started 8 weeks after the starting of anti-fungal therapy to minimise any potential Immune-Reconstitution-Inflammatory-Syndrome (IRIS) related complications. Repeat urine cultures done 3 months later were normal with complete resolution of cryptococcuria.

**Fig. 1:**
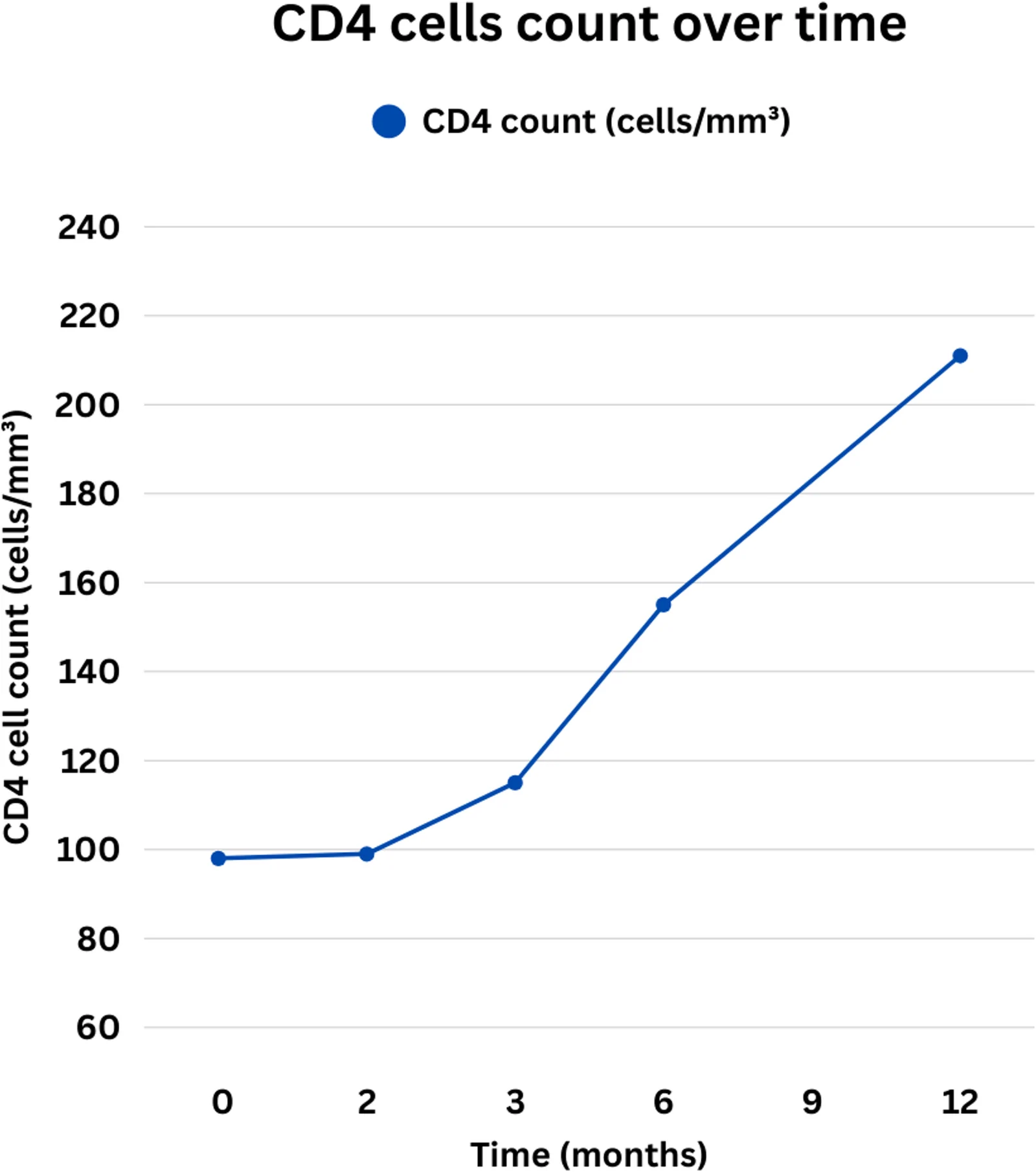
CD4 cell count over 1 year follow-up: Rising trend of CD4 cells over a year; note the CD4 cells did not rise significantly till anti-retroviral therapy was initiated and their values (cells/mm³) were 98 at presentation, 99 after 2 months, 115 after 3 months, 155 after 6 months, 183 after 9 months and 211 after 12 months follow-up

After 12 months, the patient has stabilised significantly (CD4 count 211 cells/mm³) while there were no lasting artefacts of this episode. Her CD4 counts were followed up regularly every 3 months and there was steady rise in trend, with no complications. (Fig-1)

## Discussion

*C. neoformans* is an encapsulated yeast with a thick polysaccharide capsule (visible as clear halos as the thick polysaccharide capsule remains unstained in stains like India ink preparation) and melanin-rich cell wall. The key virulence factors are the capsule which inhibits phagocytosis, and melanin which provides protection against oxidative stress. Typically, infection begins by inhalation of spores, establishment of a latent pulmonary focus, while in immunocompromised hosts the fungus can disseminate hematogenously, often involving the central nervous system [3–5]. 

Although the use of Highly-Active-Antiretroviral-Therapy has significantly lowered the incidence of cryptococcosis, the estimated global burden of HIV associated cryptococcal meningitis still approximates 1 million cases annually worldwide [6]. While this number is staggering, *Cryptococcus* most commonly manifests as meningitis or pneumonia, and Cryptococcal peritonitis remains an extremely rare manifestation. A literature review identified 61 cases of cryptococcal peritonitis over six decades, majority of which were associated with primary immune deficiency; 19.7% of them being HIV/AIDS, with liver cirrhosis (45.9%) leading the list followed by End-stage renal disease on peritoneal dialysis (24.6%) [2]. Although concomitant CNS and urinary involvement was reported in 16.4% and 8.2% of cases respectively, peritoneal disease in these cases are recognised only after disseminated infection has already been established or alongside concurrent neurological manifestations [2]. In contrast, our patient presented with an acute surgical abdomen suggestive of perforated appendicitis, without headache, meningeal signs, or other evidence of CNS involvement at admission. Cryptococcal infection was first identified from appendiceal and peritoneal histopathology, and diagnosis of cryptococcal meningitis became clinically apparent only on postoperative day 3. This temporal sequence—clinically overt peritonitis preceding the diagnosis of cryptococcal meningitis—distinguishes our case from most previously reported cases, where CNS disease was either already recognised, diagnosed concurrently with peritonitis, or formed part of established disseminated infection. Since this review was published, very few cases of cryptococcal peritonitis, particularly in HIV-positive patients have been reported. One such case reported a cryptococcal ventriculoperitoneal (VP) shunt infection, placed for hydrocephalus, resulting in concurrent peritonitis and meningitis [7]. Therefore, cryptococcal peritonitis should be included in the differential diagnosis of peritonitis, especially in patients with concomitant immunocompromised states. Our report also underscores that abdominal cryptococcosis can precede CNS disease, so clinicians should consider early systemic evaluation even when peritonitis seems primary.

Clinical manifestations of cryptococcal peritonitis are very nonspecific and often mimics spontaneous bacterial peritonitis [3]. Patients typically present with fever, abdominal pain or distension, and worsening ascites. In many cases, in ascitic fluid analysis neutrophils predominate initially (just like Spontaneous Bacterial Peritonitis), but a higher lymphocyte percentage than a typical bacterial peritonitis is often a subtle clue. If bacterial cultures remain negative after 48 h (and particularly if the patient is not improving on empirical broad-spectrum antibiotics), it should prompt suspicion of an underlying fungal peritonitis [3, 8]. 

Diagnosis of cryptococcal peritonitis begins with prompt diagnostic paracentesis in at-risk patients. Ascitic fluid should be examined with appropriate fungal stains, cryptococcal antigen testing, and culture [3, 9]. Once *Cryptococcus* is identified at any extra-pulmonary site, current guidelines recommend systematic evaluation for disseminated disease irrespective of neurological symptoms [1, 10, 11]. This evaluation should include serum cryptococcal antigen testing, preferably with quantitative titers and lumbar puncture to assess for CNS involvement, as subclinical cryptococcal meningitis may be present even in neurologically asymptomatic patients. Early identification of dissemination is essential because CNS involvement significantly influences prognosis, therapeutic strategy, and duration of treatment. Accordingly, identification of intra-abdominal cryptococcosis should prompt immediate investigation for systemic disease. Investigations for disseminated disease should include: (1) a lumbar puncture with CSF cryptococcal antigen and culture, (2) quantitative serum cryptococcal antigen testing to estimate disease burden, (3) blood cultures, and (4) appropriate imaging [1, 10, 11]. 

One of the major diagnostic challenges in this case was the patient’s initial presentation as a classical acute surgical abdomen rather than features suggestive of systemic opportunistic infection. Although HIV seropositivity was identified during pre-operative evaluation, the patient had no prior history of immunosuppression, no documented opportunistic infections and lacked neurological symptoms, chronic pulmonary symptoms, constitutional weight loss, headache, altered sensorium, focal neurological deficits, or signs of raised intracranial pressure at presentation. Instead, she demonstrated localized right iliac fossa tenderness with guarding, rebound tenderness, pelvic free fluid on ultrasonography-findings suggestive of perforated appendicitis with intra-abdominal sepsis. These findings strongly favored a surgical pathology requiring urgent intervention. The pre-operative HIV positivity and the acute surgical abdomen were viewed as isolated events at the time of presentation.

Furthermore, cryptococcal peritonitis is exceptionally rare, particularly in young HIV-positive patients without cirrhosis, peritoneal dialysis, or previously established disseminated fungal disease [2]. In most clinical settings and also in our case, acute appendicitis and bacterial intra-abdominal infection are considered more likely diagnoses than opportunistic fungal peritonitis. The absence of any neurological symptoms at admission also reduced the clinical suspicion for disseminated cryptococcosis, if at all. Consequently, the diagnosis became apparent only after histopathological examination of the appendiceal and omental specimens demonstrated encapsulated yeast forms consistent with *C. neoformans*. The subsequent onset of headache and neck stiffness on postoperative day 3, and arrival of histopathological reports prompted investigation for central nervous system involvement, ultimately confirming cryptococcal meningitis. This sequence highlights how atypical organ involvement may obscure the diagnosis of cryptococcosis, even in patients subsequently found to have advanced HIV infection. Retrospectively, the patient’s low CD4 + count of 98 cells/mm³ was a key risk factor, but it was unknown at presentation (when later revealed, eventually led us to broaden our differential diagnosis to include opportunistic infections).

**Fig. 2:**
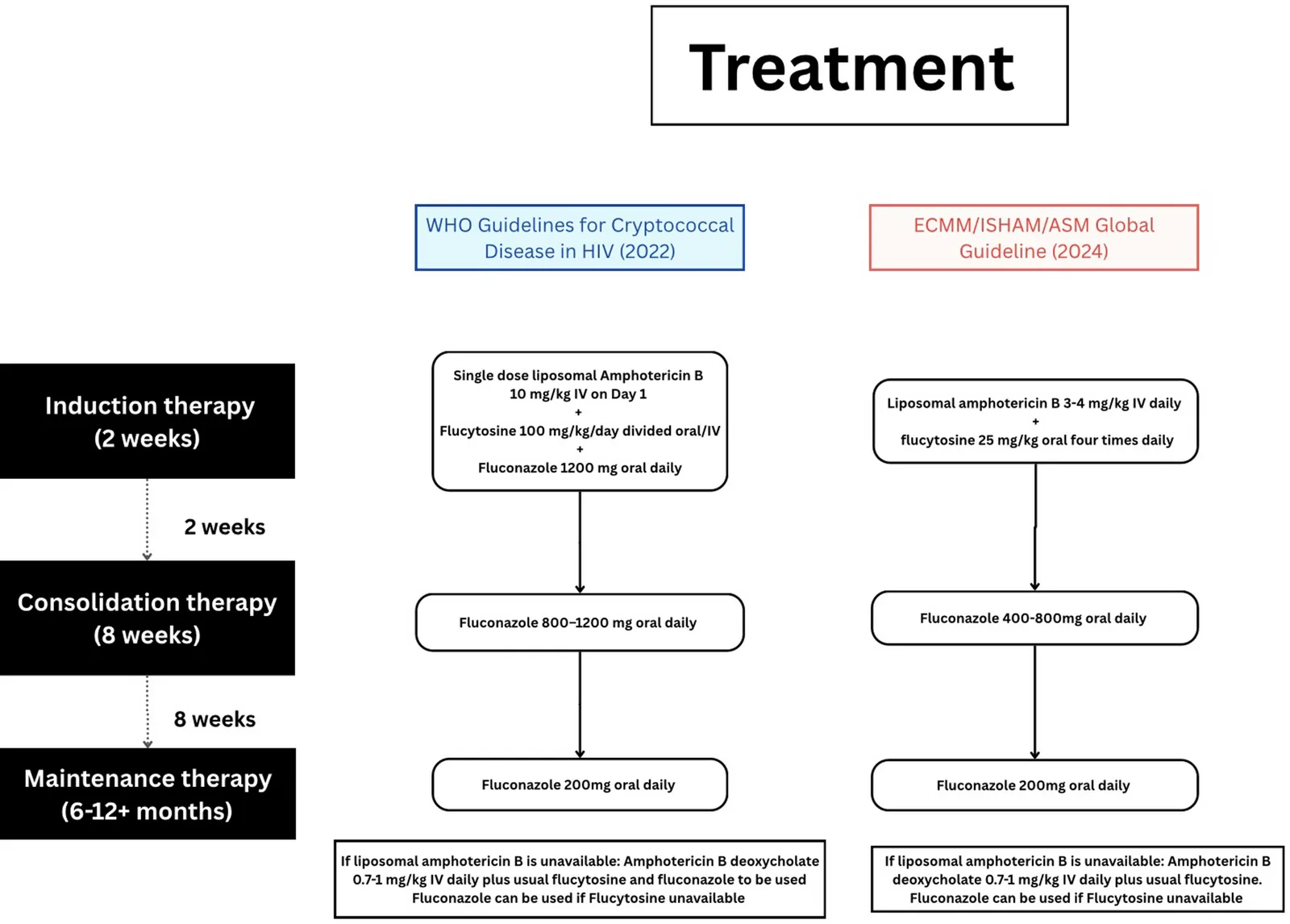
Treatment protocol for Cryptococcal meningitis and disseminated cryptococcosis: Both WHO guidelines for Cryptococcal Disease in HIV 2022 and ECMM/ISHAM/ASM Global Guideline 2024 are listed [1, 10]

Current treatment recommendations for cryptococcal meningitis and disseminated cryptococcosis are outlined in two major guidelines. (Fig. 2) [1, 11] Both recommend an initial 2-weeks induction phase. If CSF cultures remain positive at the end of induction therapy, reinitiation of an additional 2-week induction course can be considered. This is followed by an 8-week consolidation phase with high dose oral fluconazole; then a prolonged maintenance phase with oral fluconazole for atleast 6–12 months until immune recovery is achieved, cryptococcal antigen becomes negative, and relapse risk is minimised. Liposomal amphotericin B is preferred, while amphotericin B deoxycholate remains as an alternative in resource-limited settings. If flucytosine is unavailable, fluconazole alone may be used, although recent evidence suggests lower resistance rates and improved outcomes when fluconazole is combined with flucytosine [1, 11]. 

Contemporary WHO and international guidelines recommend flucytosine-containing regimens whenever available because they are associated with more rapid cerebrospinal fluid fungal clearance and improved survival outcomes (Fig. 2). However, these same guidelines acknowledge that flucytosine remains unavailable in many low and middle-income settings and provide amphotericin B plus high-dose fluconazole as an acceptable alternative regimen when flucytosine is unavailable as is the case in our institution and most parts of our country [1, 11]. Given the life-threatening nature of disseminated cryptococcosis and cryptococcal meningitis, antifungal therapy was initiated immediately rather than delaying treatment while attempting to procure flucytosine.

This limitation reflects a broader challenge in many low and middle-income countries, specially in most parts of Asia and Africa, where access to flucytosine remains inconsistent despite its inclusion in international treatment guidelines. Recent reports from international fungal disease networks have highlighted persistent supply constraints and limited availability of flucytosine across Asian regions, leading clinicians to rely on alternative induction regimens when necessary [12–14]. Fluconazole 800 mg was started for the first 2 days and then increased to the standard high-dose regimen of 1200 mg after initiation of Amphotericin B deoxycholate regimen as the patient showed transiently elevated aspartate transaminase (141 U/L) and alanine transaminase (135 U/L) for the first two days before coming back down to normal. High-dose fluconazole was initially deferred to avoid potential hepatotoxicity in our patient already recovering from peritonitis [15]. Following the start of antifungal treatment patient remained hemodynamically stable throughout hospitalisation, had no evidence of markedly elevated intracranial pressure, no focal neurological deficits, and demonstrated progressive clinical improvement.

Although India ink staining remained positive at two weeks, re-initiation of induction therapy was withheld because the patient showed rapid clinical recovery without any neurological deteriorations, supporting continuation of planned consolidation and maintenance therapy according to standard guidelines. Therefore, while the induction therapy deviated from the preferred regimen, this initial change was effective and did not affect the outcome as evidenced by the subsequent uncomplicated follow-up period, no lasting artefact of this episode and progressive immune reconstitution without relapse over the next 12 months. (Fig-1)

In conclusion, cryptococcal peritonitis is a rare but potentially life-threatening manifestation of disseminated cryptococcosis. It should be considered in immunocompromised patients with ascites who fail to respond to standard antibacterial therapy. Atypically, it can present initially as peritonitis and later progress to more commonly encountered meningitis variant, thus its important to be wary of dissemination. Additionally, although lymphocytic CSF in India primarily points towards Tuberculosis, *Cryptococcus* and Listeria meningitis should be kept as differentials specially in immunocompromised patients.

## Supplementary Information

Below is the link to the electronic supplementary material.

Supplementary Material 1

## Data Availability

No datasets were generated or analysed during the current study.

## Declarations

### Patient perspective

When I first developed fever and headache, I thought it was a cold, but it stayed for a long while. I started developing pain in my abdomen and took antacids. But suddenly one day the pain really increased, and it was difficult to move, I could barely stay awake. My family members brought me to the hospital and doctors told me I had to get operated urgently; they needed to remove liquid from my abdomen. I was scared as I was not prepared for such things, but my family and doctors calmed me down. They told me I had HIV, but I realised it much later, and when I did, I was devasted thinking I cannot live, but more what everyone will think of me. I did not want to speak with my family, but the doctors helped us talk after one day. I was relieved when my family supported me but there were still some doubts at that time. I’m now going to therapy and taking medicine regularly to live better.
