# Supplementary material for: Cryptic clues from an acute abdomen: a case report of peritonitis turning into disseminated cryptococcosis
Source: BMC Infect Dis. 2026 Jul 16;26:1797. doi: 10.1186/s12879-026-13871-w (PMC13659984; doi:10.1186/s12879-026-13871-w)
Supplement: Supplementary file 1 [file 12879_2026_13871_MOESM1_ESM.docx]

**A**

**B**

**Supplementary Fig-A	Supplementary Fig-B**

**C**

**D**

**Supplementary Fig-C	Supplementary Fig-D**

Supplementary Figure-A: **H&E staining on omental specimen** showing transmural inflammation with many oval fungal infections, some showing clear halos (unstained parts, as seen in negative staining) suggestive of *Cryptococcus*

Supplementary Figure-B: **Special stain- Grocott methenamine silver positivity**, showing black-stained round-to-oval yeast cells with surrounding clear halos (unstained parts, as seen in negative staining), suggestive of *Cryptococcus*.

Supplementary Figure-C: **Special stain- Periodic Acid-Schiff positivity**, showing magenta-colored yeast cells with prominent polysaccharide capsule(halo)

Supplementary Figure-D: **Periodic Acid-Schiff** **stain positive in urine sample** with prominent surrounding halos (unstained parts, as seen in negative staining), suggesting cryptococcuria
